# Case Report: A case of suprapiriform ureterosciatic hernia with ureteral incarceration treated by total extraperitoneal laparoscopic technique

**DOI:** 10.3389/fsurg.2026.1867501

**Published:** 2026-10-08

**Authors:** Dan Luo

**Affiliations:** Department of General Surgery, Mianyang Central Hospital, Affiliated with Electronic Science and Technology University Medical College, Mianyang, Sichuan, China

**Keywords:** hernia, pelvic floor hernia, sciatic hernia, surgery, totally extraperitoneal approach

## Abstract

Sciatic hernias (SH) are one of the rarest forms of pelvic floor hernia. Suprapiriform sciatic hernia (SSH) is the most common type among them. We present the case of a male patient with a preoperative contrast enhanced CT urogram suggesting left ureteral incarceration due to a left ureterosciatic hernia. With the combined efforts of the general surgery and urology departments, we performed a tension-free repair of the ureterosciatic hernia using a total extraperitoneal laparoscopic approach (TEP). Additionally, the left ureter is released from the hernia sac and a double J ureteral stent placement were accomplished via transurethral ureteroscopy. Intraoperative diagnosis confirmed left suprapiriform ureterosciatic hernia with left ureteral incarceration. The double J ureteral stent was removed one month after surgery and follow up continued for 3 months, during which time the patient showed no signs or symptoms of recurrence. Therefore, total extraperitoneal laparoscopic exploration and repair can be considered a favorable treatment modality for symptomatic suprapiriform ureterosciatic hernia and other types of sciatic hernia. Especially in view of the advantages of minimal trauma, rapid recovery, better visual field, convenient observation of surrounding anatomical structures, and improved operation maneuverability.

## 1 Introduction

SH account for about 0.01% of all abdominal hernias (1) and are a very rare type of pelvic floor hernia. There are three main types of sciatic hernia: suprapiriform hernia, infrapiriform hernia, and spinotuberous hernia with SSH being the commonest. Contents found in ischiatic hernias usually include ovary, small intestine, ureter, bladder, colon, Meckel's diverticulum, appendix, benign and malignant soft tissue tumors, etc (2). Among them, the percentage of cases with ureteral involvement is about 25% (3). Complete or partial entrapment of the ureter through the hernial orifice can cause hydroureter and hydronephrosis, leading to urinary system infections, pyelonephritis, urinary sepsis, obstructive uropathy, renal insufficiency and other critical conditions. Therefore, symptomatic ischiatic hernias with ureteral incarceration require active and prompt treatment. Most cases reported in the literature involved either conservative treatment with double J ureteral stent insertion or open surgery with greater trauma. Cases of minimally invasive repair via laparoscopic or robotic approaches have been very few. A review of the literature revealed even fewer reports of laparoscopic repair using total extraperitoneal techniques without entering the abdominal cavity or breaching the peritoneum. Here, we report a case of successful total extraperitoneal laparoscopic repair for a ureterosciatic hernia of suprapirifom type, and a retrospective review of the relevant literature.

## 2 Case description

### 2.1 Preoperative examination and preparation

A 76-year-old male with a BMI of 22.37 was admitted to the urology department of our hospital due to “repeated left lower abdominal distention and pain for 1 year with recent aggravation for 10 days”. He underwent contrast enhanced CT urography and reports suggested a left ureterosciatic hernia most likely through the greater sciatic foramen, associated with moderate to severe hydroureter and hydronephrosis of the left side (Figures 1A,B). Urological ultrasound: The upper segment of the left ureter was dilated with moderate hydronephrosis of the left kidney, the prostate was moderately enlarged with calcifications. Laboratory tests: Urinalysis and Urinary system tumor markers were all within normal ranges. Abnormal indicators of renal function were: SerumCr 114.6umol/L, eGFR(Cr) 53.2 mL/min/L, eGFR(Cr + CysC) 47.9 mL/min/L, C1q 239 mg/L, CysC 1.48 mg/L.Differential FBC only showed following abnormalities: Lymphocyte count 0.91*10^9/L, Eosinophil count 0.72*10^9/L, Percentage of Lymphocytes 11.7%, Percentage of Neutrophils 75.5%, and Eosinophil percentage 9.2%.He was in good general health, denied any history of coronary heart disease, hypertension, diabetes, chronic bronchitis or any other chronic medical conditions. However, there was history of left ureteral stent placement, performed in another hospital about a year ago following a diagnosis of “left ureteral stenosis”. The physical examination was unremarkable with normal vital signs. Abdominal and genitourinary system examinations showed no palpable renal or bladder masses, no tenderness on superficial or deep palpation, no renal angle tenderness and no renal bruits. The initial diagnosis was a left ureterosciatic hernia in the greater sciatic foramen with left ureteral incarceration. There were no absolute contraindications in preoperative assessment. Following discussion between the departments of general surgery and urology, the decision was made to perform a total extraperitoneal laparoscopic surgery for exploration and mesh repair of the left greater sciatic foramen hernia, release of the incarcerated left ureter, and placement of a left ureteral stent via transurethral ureteroscopy. The patient's informed written consent has been obtained for surgery under general anesthesia and release of case details and related images.

**Figure 1:**
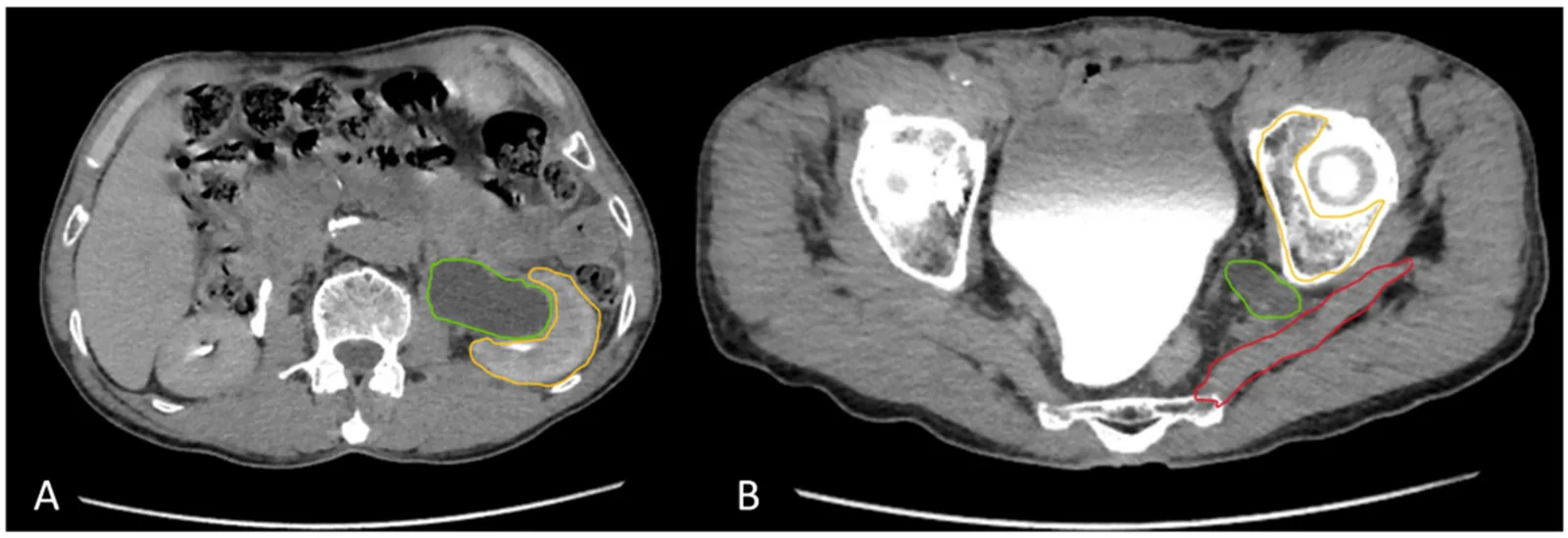
Contrast enhanced CT of urinary system. **(A)** indicating left ureteral hernia in the greater sciatic foramen with ureter going above the plane level and moderate to severe hydroureter and hydronephrosis (encircled in green is the left ureter with severe hydroureter and yellow is the dilated left kidney with hydronephrosis). **(B)** showing hernia of the left ureter in greater sciatic foramen, with an above-plane ureter and moderate to severe hydroureter. (Green indicates the ureter, yellow indicates the ischial body and red indicates the piriformis muscle).

### 2.2 Surgical procedure

The patient was intubated under general anesthesia. First placed in Trendelenburg position, later followed by combined lithotomy position, slightly to the right. A 12 mm trocar was placed into the posterior margin of rectus abdominis muscle through an infra-umbilical incision and used as the camera port for observation. After adequate insufflation was established in the extraperitoneal space, two 5 mm trocars were placed between the umbilicus and the pubis as working ports for the surgeon. (The trocar placement method was identical to that used in the TEP technique for midline hernias, see Figure 2A). First the anterior vesical space was entered, and careful dissection and separation was done, then the left ilioinguinal space was entered. The left ilioinguinal space was further separated at the lateral margin of the bladder and on the surface of obturator fascia till the iliac vessel bifurcation, and the greater sciatic foramen was visualized and exposed. The left ureter was found to be contorted at the suprapiriform space and attached to the left superior gluteal artery and nearby structures. The ureter was carefully dissected out and gradually released and returned to the normal position (Figures 2B,C). At this time, the transurethral ureteroscope was inserted and a guidewire was introduced to the left ureter and the ureteral orifice was expanded. Then a ureteral catheter was advanced over the guidewire up to the renal pelvis and a double J 4.7# ureteral stent was placed in the left ureter (Product name: ureteral stent, specification and model: 788426). After proper placement and deployment of the stent the guidewire and ureteroscope were removed. Then structures surrounding the hernia sac were further separated and the hernia mesh was placed carefully. The mesh does not require any fixation. (Product name: hernia repair mesh, Model: LPPMT, specification: 12*16 cm, Company: Beijing Tianzhu Changyun Medical Technology Co., LTD.) (Figure 2D). Afterwards no active bleeding confirmed, insufflation desusfflated, and the incisions were closed, completing the operation.

**Figure 2:**
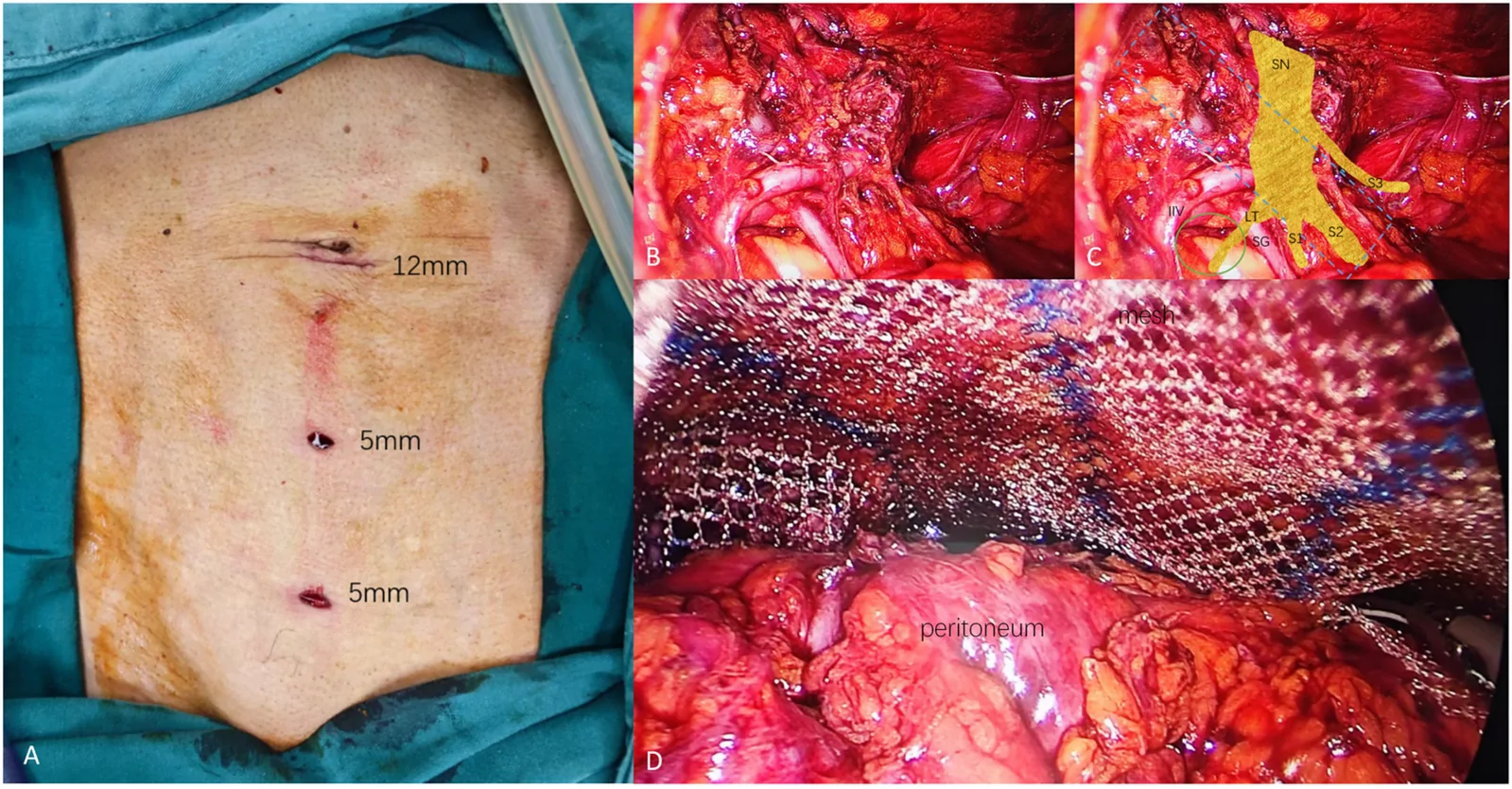
Imaging during surgery. **(A)** Three ports for trocar placement were established between the umbilicus and the pubic bone (12 mm, 5 mm, 5 mm trocar were inserted from top to bottom). **(B)** The operative field around the greater sciatic foramen after left ureter release and ureteral stent placement. **(C)** Anatomical labeling of Figure B. (Green circle shows the region of the defect that functioned as the hernial orifice, The blue dotted line represents the piriformis muscle outline, SN:sciatic nerve,LT:lumbosacral trunk,S:spinal nerve,IIV:internal iliac vessels, SG:the superior gluteal arteries and veins). **(D)** Placement of the hernia repair mesh.

### 2.3 Postoperative management and follow-up

Postoperative abdominal xrays showed proper placement of the double J ureteral stent and confirmed proper functioning of the left urinary system (Figure 3). After surgery, on day one the patient independently mobilized out of the bed, normal diet was resumed on day two, and he was discharged after urinary catheter removal on day three. The patient was carefully followed up and the double J ureteral stent was removed one month post-op. On 3 month follow up visit, he was free of any signs or symptoms. To be honest, we did not conduct any ultrasounds or imaging tests later on to rule out asymptomatic recurrence of hydronephrosis from recurrent hernia or stricture. After being discharged from the hospital, this patient did not experience any further discomfort and did not undergo any further urinary system ultrasound examinations.

**Figure 3:**
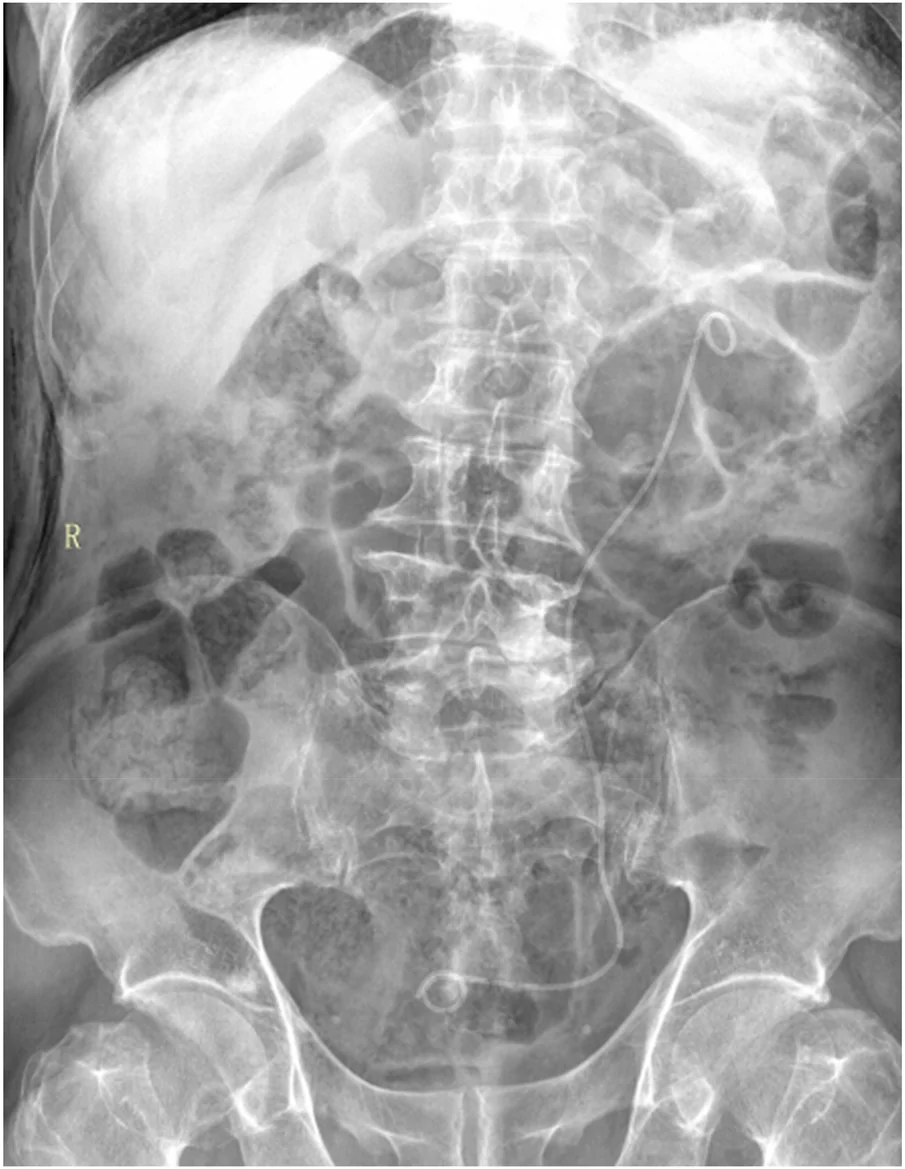
Post-operative image.postoperative (post op day 1) abdominal radiograph showing proper placement of the double J ureteral stent.

## 3 Discussion

There are three types of pelvic floor hernia; obturator hernia, perineal hernia and sciatic hernia (4). SH are the rarest among all three and account for about 0.01% of all abdominal hernias. They are reported to be more common in women (1). It was first described by Papen in 1750 and observed and recorded by Verdier in 1753 (5). There are currently only about 100 cases reported in medical literature worldwide. The sciatic foramen is formed by the sciatic notch at the upper margin and the sacrospinous ligament and the sacrotuberous ligament at the lower margin of the pelvis. The sacrospinous ligament separates the greater sciatic foramen from the lesser sciatic foramen. The greater sciatic foramen is further divided into two parts by the piriformis muscle, the suprapiriform space and the infrapiriform space. This ultimately leaves three potential protrusion sites for hernias. These 3 SH are namely: suprapiriform hernia, infrapiriform hernia, and spinotuberous hernia with suprapiriform hernia being the commonest type. Common ureteral hernia sites are the inguinal and femoral canals, and herniation into the sciatic foramina is extremely rare (6). It has been reported in the literature that the incidence of sciatic hernia containing a ureter is about 25% out of all SH (3). In 1947, Swedish radiologist Ake Lindbom discovered and reported the first case of ureterosciatic hernia, which was subsequently labeled as Lindbom hernia. Ake Lindbom's characterization of the typical radiological sign of the condition, the “curlicue sign”, contributed significantly to the identification of this hernia (7).

The mechanism by which the SSH occurs remains unclear. But identified risk factors include female gender, wider pelvic opening, history of multiple pregnancies, advanced age, sudden weight loss, constipation, chronic intra-abdominal high pressure, history of benign and malignant pelvic tumors, history of other types of pelvic floor hernias, history of hysterectomy or bladder suspension, history of pelvic trauma, history of long-term indwelling IUD resulting in pelvic actinomycosis, history of pelvic abscess, and ischium intrauterine membrane heterotopia, primary piriformis syndrome, etc (1, 4, 6–8).. Potential causes include partial pelvic fascia loss, connective tissue loosening, atrophy and weakness of piriformis muscle, adhesions of piriformis muscle, or congenital malformation and abnormal development (4, 6, 7, 9–11). Our patient is a male with no significant past history. But he has a low BMI value, and low body weight has been shown to have correlation with this type of hernia. It has also been proposed that suprapiriform ureterosciatic hernia is further developed by extension of the defect at the pelvic wall fascia along the superior gluteal artery, inferior gluteal artery, or pudendal artery (7). In addition, some scholars have pointed out that the left ureter is anatomically longer than the right ureter and because of that carries a higher risk for herniation (6). Also, in patients with chronic constipation, more pressure is accumulated on the left side of the colon which in turn increases the push on the left ureter. Therefore, ureterosciatic hernias are more likely to occur on the left side (9), with an incidence of about 88% out of all ureterosciatic hernias (3). In our case the same could be observed.

It is difficult to diagnose a sciatic hernia solely from the patient's signs and symptoms. SH can contain a variety of structures including ovary, small intestine, ureter, bladder, colon, Meckel's diverticulum, appendix and benign and malignant soft tissue tumors. The corresponding clinical manifestations can be lateral abdominal, central abdominal, pelvic, lower back or thigh region associated without any other specificity. Complications may also vary due to the different contents of the hernia. Depending on the content of the hernia, the degree of herniation, incarceration and presence or absence of hernia strangulation, outcome also changes. It could present as a hip mass, a small intestinal obstruction, septicemia, hydronephrosis, buttock pain with or without sciatica etc (2, 4). The inherent spontaneous reducibility of SH (7) also explains the intermittent nature of symptoms, such as the chronic on and off abdominal pain our patient suffered for over a year. However, when the hernia contents are ureters, physical examination is often accompanied by renal angle tenderness. Intensity of renal angle tenderness could be related to the degree of hydronephrosis (10). In this case, the main manifestation and the presenting complaint was left lower abdominal distension and pain, but physical examination was unremarkable including no renal angle tenderness. Persistent hydronephrosis can further lead to urinary tract infections and urinary sepsis (6). Fortunately, our patient did not have any such complications even with history of hydronephrosis lasting for nearly one year. At present, the diagnosis of incarcerated ureterosciatic hernia is almost always made by preoperative imaging investigations (4). Radiographic imaging plays a crucial role in determining the presence of ureteral obstruction and identifying other possible causes (7). Imaging tests used to detect ureterosciatic hernias include ultrasound, computed tomography (CT), contrast urography and MRI (3). A curvelike structure in the greater or lesser sciatic foramen areas seen on radiogrpahic images in suspected cases is suggestive of ureteral prolapse (3). In earlier days, retrograde pyelography and intravenous urography (IVU) were used to show the presence of ureteral loops through the sciatic foramina, with sensitivity of 66%–87% and specificity of 92%–94%. However, with the advent of advanced CT scans, which provide superior information compared to IVUs, practice has shifted more towards the utilization of CT IVU scans as the primary imaging mode (7). This case was also suspected initially through help of preoperative contrast enhanced CT urography.

In the early days of sciatic hernia treatment, transgluteal or transabdominal open surgery was the common approach, with the defect being closed with sutures. In more challenging cases, muscle or fascia approximation and gluteal muscle flaps were employed to close the defect (4). With advancements in technology, modern treatments now include options such as close observation without intervention, nephrostomy, ureteral stent placement, transvaginal manual reduction, laparoscopic surgery, and robot-assisted surgery (3, 6). Observation without intervention is only recommended for stable asymptomatic patients who are incidentally discovered during routine checkup or assessments. Nephrostomy is mainly used in patients with severe hydronephrosis and obvious urosepsis who need prompt relief of the urinary tract obstruction. Ureteral stenting is one of the most widely used treatment methods. The procedure is relatively simple and minimally invasive, and it can be used as temporary treatment in high-risk surgical patients who cannot be operated on before optimization. However, it should be emphasized that stenting is only a symptomatic treatment modality, not a curative treatment for obstruction due to a sciatic hernia. Long-term follow-up is necessary, as stenting carries a higher recurrence risk compared to surgical intervention. Our patient had previously undergone ureteral stent placement at another hospital about a year ago but experienced recurrent abdominal pain again after stent removal, indicating that the ureterosciatic hernia had always existed causing his symptoms. Treatment of ureterosciatic hernias with surgery includes releasing the ureteral hernia segment, reorganizing the connective tissue and closing with sutures or repairing the defect with a hernia mesh. These procedures always be performed through open (transabdominal or transgluteal) approaches (2). It took 52 years since publication of Lindbom's first case report on sciatic hernia, for the first publication of a laparoscopic approach to sciatic hernia repair, which was done in 1999 (7). now. Laparoscopic approach to repair of SH facilitates detailed and less traumatic visualization of the deep pelvis and allows for easier identification and effective dissection of surrounding anatomical structures and fascial layers located near and around the sciatic foramina (1). Since Gee et al. reported on the first laparoscopic repair of sciatic hernia in 1999, the number of reported cases of laparoscopic repair for these hernias still remain in double digits (12). In our case we used a total endoscopic extra-peritoneal technique, which does not involve entering the peritoneal cavity, yet provides a better view of the surrounding anatomy. In addition, the preperitoneal patch placement reduces possible mesh related complications and the need for patch fixation. The literature reports that there was only one patient who underwent the TEP surgical procedure for this disease (13). However, dissection in this region is challenging due to its proximity to the branches of the internal iliac vessels, the superior gluteal arteries and veins, as well as the sacro-lumbar trunk and the superior gluteal nerves, among other important anatomical structures.

Preoperative discussions with urologists and anorectal and pelvic floor surgeons gave us further insight into the complex anatomy and surrounding structures. In our patient the tortuous ureter and the surrounding vessels were particularly difficult to identify and separate, therefore we first proceeded with placement of the ureteral stent via transurethral ureteroscopy before confirming the identity of the left ureter and continuing with careful dissection and gradual release. In general, laparoscopic mesh repair of ureterosciatic hernia not only allows better correction anatomically, but also provides better outcome physiologically and symptomatically, with all the advantages of minimally invasive techniques such as faster recovery, less pain and better cosmetic results.

However, the debate regarding the optimal treatment modality for symptomatic ureterosciatic hernia is still ongoing. Some studies advocate immediate surgical repair, whether open, laparoscopic or robot-assisted, while others opt for temporary ureteral stent placement before considering final surgical repair or long-term maintenance of a ureteral stent (7). Based on our clinical experience, we believe in utilizing the most suitable treatment modality for the patient depending on their general condition. For asymptomatic patients discovered incidentally close observation without intervention can be considered. In elderly patients with multiple comorbidities or those in septic shock with unstable vital signs who are not candidates for surgery, retrograde stenting or nephrostomy offer temporary safe and effective treatment. For symptomatic patients, with no contraindications for laparoscopic surgery, a total extra peritoneal laparoscopic mesh repair is a better option. This procedure requires skills similar to those used in standard TEP hernia repair, making it feasible for most experienced laparoscopic hernia surgeons (1). However, the closure force required for sciatic hernia repair differs significantly from that of inguinal hernia repair (4), so attention should also be paid to the selection of the hernia repair mesh. Given the limited research in this area, further studies and discussions are warranted.

## 4 Conclusion

Ureterosciatic hernia is a rare disease, so the literature worldwide is very limited, yet it poses significant risks due to its potential to cause ureteral obstruction (14). In our case we successfully relieved the incarceration of the ureter through total extra-peritoneal laparoscopic surgery and repaired the hernia defect at the suprapiriform space of the greater sciatic foramen with a hernia mesh. This approach offers the advantages of minimal trauma, easier operation, rapid recovery and better prognosis. At the 3-month follow-up, our patient showed no signs or symptoms of recurrence, suggesting successful treatment; however, longer observation is necessary to confirm the long-term effectiveness. Based on our experience, we recommend laparoscopic mesh repair for suprapiriform ureterosciatic hernias and other types of sciatic hernias.

## Data Availability

The raw data supporting the conclusions of this article will be made available by the authors, without undue reservation.
